# Guano-Derived Nutrient Subsidies Drive Food Web Structure in Coastal Ponds

**DOI:** 10.1371/journal.pone.0151018

**Published:** 2016-03-08

**Authors:** Salvatrice Vizzini, Geraldina Signa, Antonio Mazzola

**Affiliations:** Department of Earth and Marine Sciences, University of Palermo, CoNISMa, via Archirafi 18, 90123 Palermo, Italy; University of Fribourg, SWITZERLAND

## Abstract

A stable isotope study was carried out seasonally in three coastal ponds (Marinello system, Italy) affected by different gull guano input to investigate the effect of nutrient subsidies on food web structure and dynamics. A marked ^15^N enrichment occurred in the pond receiving the highest guano input, indicating that gull-derived fertilization (guanotrophication) had a strong localised effect and flowed across trophic levels. The main food web response to guanotrophication was an overall erosion of the benthic pathway in favour of the planktonic. Subsidized primary consumers, mostly deposit feeders, switched their diet according to organic matter source availability. Secondary consumers and, in particular, fish from the guanotrophic pond, acted as couplers of planktonic and benthic pathways and showed an omnivorous trophic behaviour. Food web structure showed substantial variability among ponds and a marked seasonality in the subsidized one: an overall simplification was evident only in summer when guano input maximises its trophic effects, while higher trophic diversity and complexity resulted when guano input was low to moderate.

## Introduction

Allochthonous nutrient subsidies across ecosystem boundaries have far-reaching consequences for ecosystem functioning due to the influence on primary production and food webs [1]. Defining a generalised ecosystem response to nutrient subsidies is not possible, because responses vary widely. In this respect, seminal modelling studies have suggested that donor-controlled nutrient subsidies into the basal levels of a recipient food web may lead to bottom-up effects, whose influence on trophic dynamics depends on the magnitude, quality, timing and type of subsidies [1–3]. It is assumed that when subsidies are low to moderate, consumers may keep feeding on autochthonous sources, while higher input may lead consumers to switch diet in favour of external sources, respectively stabilising or destabilising food webs [2]. Additionally, empirical studies have highlighted that in donor-controlled systems, resource partitioning may be an adaptive response of consumers which may result in parallel alternative pathways and /or a high degree of omnivory [4,5].

Guano input by seabirds is a widespread phenomenon that represents a nutrient subsidy in recipient systems. Indeed, seabirds are mobile organisms acting as biovectors across coastal boundaries and affecting the functionality of recipient ecosystems through abundant release of nutrients [6–9]. High concentrations of organic and inorganic nutrients in guano [3,10,11] fertilise recipient food webs through the enhancement of primary production with consequences that ramify throughout trophic levels [12]. Moreover, seabird nutrient subsidies increase the nutritional quality of primary producers in terms of higher nutrient concentration and palatability [3], allowing larger consumer populations and longer food chains. However, they also trigger higher phytoplanktonic productivity, determining detrimental effects on benthic communities similar to those associated with severe eutrophication [13]. While seabird-induced increase in primary and secondary productivity has been observed worldwide [3,11], trophic responses to seabird-nutrient subsidy have been less thoroughly examined, especially in coastal marine areas, which are preferential stop-over and nesting sites for a wide variety of seabirds [14].

Carbon and nitrogen stable isotope analysis is a powerful tool to discriminate autochthonous *vs*. allochthonous input and to study organic matter pathways and food web structure in coastal systems [15–17]. Ornithogenic nitrogen is ^15^N-enriched due to the isotopic fractionation occurring during volatilisation of the ammonia from seabird guano [18]. Consequently, the stable nitrogen isotope ratio (δ^15^N) is a proxy for ornithogenic influence [7,9,18–20]. Sediment, water and primary producers close to bird colonies are ^15^N-enriched, reflecting the intensity of avian input in the system [9,19,21]. In addition, consumers feeding on isotopically enriched organic matter sources reflect the high δ^15^N of ingested food [6,20,22]. Indeed, carbon and nitrogen isotopic compositions of consumer tissues are functions of the δ^13^C and δ^15^N of the food resource exploited and of the isotopic fractionation (0–1‰ for δ^13^C; 2–4‰ for δ^15^N) occurring between trophic levels [23–26]. Therefore, stable isotopes are effective tools to assess the contribution of seabird-derived nutrient subsidies in recipient systems and their influence on subsidized food webs.

**Fig 1:**
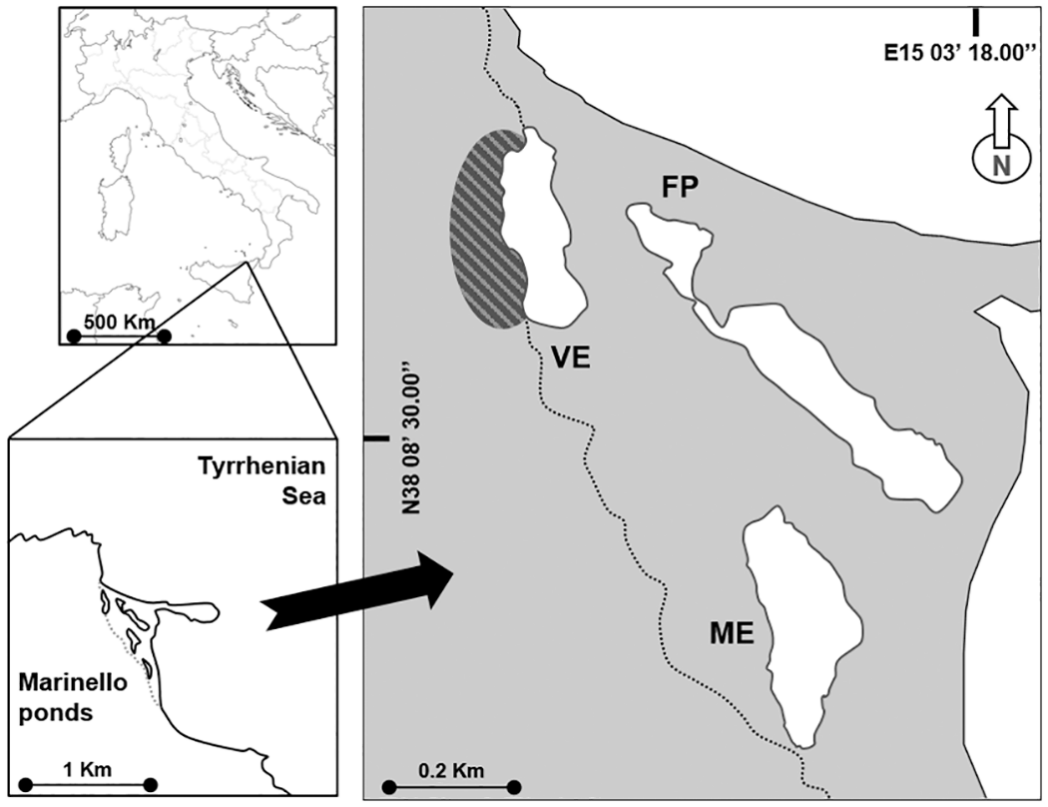
The study area of the Marinello ponds. The ponds studied were Verde (VE), Fondo Porto (FP) and Mergolo (ME), at increasing distance from the gull colony indicated by the striped oval.

The coastal system of the Marinello ponds (north-eastern Sicily, Italy, Mediterranean Sea, Fig 1) receives allochthonous input of guano from a colony of yellow-legged gulls (*Larus michahellis*). Due to the small size of the ponds (1.3–2.5 ha) and the difference in the amount of seabird guano each pond receives, the area is a suitable field laboratory for the study of the effects of guano-derived nutrient subsidy on food webs. We addressed the following question: does food web structure and dynamics differ along a gradient of seabird guano input? We expected that increased phytoplanktonic primary productivity driven by guano fertilization [9] should i) modify the trophic role of basal organic matter sources for consumers, amplifying the importance of planktonic pathways; ii) enhance the diet switch of primary consumers in favour of subsidized sources; and iii) increase omnivory, trophic diversity, redundancy and the number of trophic levels. The potential effects of guano input on food webs was evaluated by comparing the isotopic values (δ^13^C and δ^15^N) of organic matter sources and consumers and the isotopic niche and metrics [27,28] in three ponds with different avian input. Bayesian mixing models allowed estimation of the proportion of sources contributing to the consumers' diet.

## Materials and Methods

### Ethics Statement

Sampling was conducted with permits from the Authority of the ‘‘Laghetti di Marinello” Nature Reserve (permit # 28599). No other permits were required.

### Study Area

The study was carried out in the coastal system of Marinello, located on the north-eastern coast of Sicily (Italy, Mediterranean Sea) (Fig 1). The Marinello system is made up of five small brackish coastal ponds (Verde, Fondo Porto, Porto Vecchio, Mergolo della Tonnara and Marinello) separated from the sea by littoral bars and free of direct freshwater discharge. In this study, three ponds were investigated; Verde, Fondo Porto and Mergolo (hereafter VE, FP and ME respectively), chosen according to their increasing distance from the overhanging cliff, which is settled by a small gull (*Larus michahellis*) colony. VE, the pond adjacent to the cliff, is characterised by high trophic status and phytoplanktonic primary productivity [9], due to guano–derived fertilization which is amplified by small pond size (1.7 ha) and shallowness (max depth: 3.0 m). Guano input is occasional in FP due to its proximity to VE, and absent in the furthest pond, ME. Likewise, the ecological response to guano input is considerably softened in FP, and negligible in ME [9]. Gulls were also recognised as biovectors of trace elements, including toxic ones (e.g. As, Cd, Hg, Pb), and their concentrations were higher in the guanotrophic pond [20,29]. Macrozoobenthic assemblages of VE also displayed distinctive features compared with the other ponds: larger and more diverse populations were observed along the shore of VE, while a clear benthic impoverishment, up to azoic conditions, was found in the central part of the pond, where guano tends to accumulate [13]. Furthermore, the most abundant taxa found in VE were stressor-tolerant to opportunistic species, giving indication of an overall detrimental effect of guanotrophication.

### Sample collection

Sampling was carried out seasonally (Autumn 2008—Summer 2009) in the three selected ponds (VE, FP, ME). Gull guano was carefully scraped from the cliff and the shores of VE. Suspended particulate organic matter (SPOM) was obtained from surface water sampled from each pond with 5 l plastic bottles. Sediment and primary producers were collected randomly in two sites (shore and bottom) for each pond, in accordance with previous studies [20,29]. Sedimentary organic matter (SOM) was obtained from surface sediment collected using PVC cores (inner diameter: 4 cm). Seagrasses (*Cymodocea nodosa*, *Ruppia cirrhosa*, *Halophila stipulacea*) and the most abundant macroalgae (*Caulerpa racemosa*, *Chaetomorpha linum*, *Cladophora* sp.) were collected by hand. Shrimps and fish were sampled using a small hand-towed trawl net (length: 3.5 m; mesh size: 3 mm). Macrozoobenthic fauna was collected by means of a 2 l Van Veen grab (penetration depth: ∼10 cm) (see [13] for more details) and was wet-sieved directly in the field (mesh size: 0.5 mm). All food web components were collected in triplicate and kept cool and dark upon arrival at the laboratory. Sampling procedures were carried out in accordance with a protocol approved by the ‘‘Laghetti di Marinello” Nature Reserve.

### Laboratory analyses

Once in the laboratory, gull guano was wet sieved through a 1000 μm net to remove coarse residuals. The first centimetre of sediment was sliced from the core surface and wet sieved at 63 μm to collect bioavailable SOM. Pre-filtered (200 μm) surface water was filtered onto pre-combusted (450°C, 4 h) Whatman GF/F filters to obtain SPOM. After species identification, macrophytes were gently scraped to remove epibionts. Fish were identified to species level, while benthic invertebrates, after being sorted from sediment, were identified to the lowest possible taxonomic level (family, genus or species) under a stereo microscope (10 to 40x). Muscle tissues and flesh were dissected from large individuals (e.g. fish, large crustaceans and molluscs) and the whole body was used for small-bodied taxa (e.g. polychaetes and chironomid larvae). Each sample was made up of pooled specimens to incorporate inter-individual variability. After being sorted, benthic fauna from VE and ME bottom site were excluded from the isotopic analysis because of the scarcity of specimens. SOM, SPOM and guano samples were analysed separately for δ^15^N and δ^13^C. Prior to δ^13^C analysis, these samples were acidified with drop-by-drop 2N HCl to remove carbonates before drying and grinding. Conversely, primary producer, invertebrate and fish samples were directly dried to constant weight at 60°C and ground into a homogeneous powder using a mortar and pestle. Isotopic analysis was performed with an isotope ratio mass spectrometer (Thermo Delta Plus XP) connected to an elemental analyser (Thermo Flash EA 1112). Isotopic values were expressed in conventional δ unit notation (as parts per mil) in relation to international standards (Pee Dee Belemnite for δ^13^C; atmospheric N_2_ for δ^15^N), following the formula: δ^X^ = [(R_sample_/R_standard_)−1] × 10^3^, where X is the stable isotope mass (13 for C and 15 for N) and R is the corresponding ^13^C/^12^C or ^15^N/^14^N ratio. Analytical precision based on the standard deviation of replicates of internal standards (International Atomic Energy Agency IAEA-NO-3 for δ^15^N and IAEA-CH-6 for δ^13^C) was 0.2 ‰ for both δ^13^C and δ^15^N.

### Data analysis

The following food web isotopic metrics were calculated using SIBER, Stable Isotope Bayesian Ellipses in R [28]: carbon and nitrogen range for organic matter sources (OM-CR, OM-NR) and consumers (C-CR, C-NR), standard ellipse area corrected for small sample sizes (SEAc), distance to centroid (CD), and mean and standard deviation of the nearest neighbour distance (MNND and SDNND) [27]. C-CR and C-NR are indicative of niche diversification at the base of the food web and trophic length respectively. SEAc gives a measure of the total trophic diversity and of the isotopic niche of a community, while CD is a measure of the average trophic diversity. MNND and SDNND are measures of density and evenness of species packing: low MNND and SDNND indicate higher trophic redundancy, i.e. many consumers with similar trophic ecology. Food chain length (FCL), namely the maximum trophic position identified in each food web, was also estimated. Following [25], the trophic level of consumers was calculated as follows: TL_consumer_ = λ + (δ^15^N_consumer_−δ^15^N_baseline_)/3.4, where λ is the trophic position of the baseline species and 3.4 is the average ^15^N-enrichment per trophic level. To account for the high temporal variability in the δ^15^N of primary producers, we used the surface-grazing snail *Hydrobia ventrosa* as temporal integrator of δ^15^N_baseline_ for the food webs studied [30]. Where *H*. *ventrosa* was not found, the averaged value of the other seasons was used. The bivalve *Loripes lacteus* was not included in the analysis because it bears endosymbiotic bacteria that cause strong isotopic depletion [31].

Bayesian mixing models were run to estimate the contribution of each organic matter source to the consumers’ diet using the software package SIAR (Stable Isotope Analysis in R) [32]. Trophic enrichment factors, TEF, were 0.4 ± 1.3‰ for δ^13^C and 3.4 ± 1.0‰ for δ^15^N, according to [25]. The most abundant OM sources were chosen as end-members and grouped into SPOM, SOM, macroalgae and seagrasses. The isotopic signatures for macroalgae and seagrasses were pooled as a unique source (Alg and Seag) as the different species had similar values (non-parametric Mann-Whitney U-test. p>0.05 in all cases). In order to identify the prevailing OM pathways characterising the Marinello food webs throughout the seasons, a principal coordinate analysis (PCO) based on the Euclidean distance matrix calculated from the Bayesian mixing model outcomes was performed using the software PRIMER v6 (PRIMER-E Ltd., Plymouth, UK). Minimum, maximum (expressed as 95th percentiles) and mean contributions of each food source to the consumers’ diet estimated using SIAR were used as variables of the PCOs, encompassing the whole variability in the estimation of food source contributions. The vectors corresponding to the mean contribution of OM sources were superimposed on each graph.

## Results

### Isotopic values and metrics

**Fig 2:**
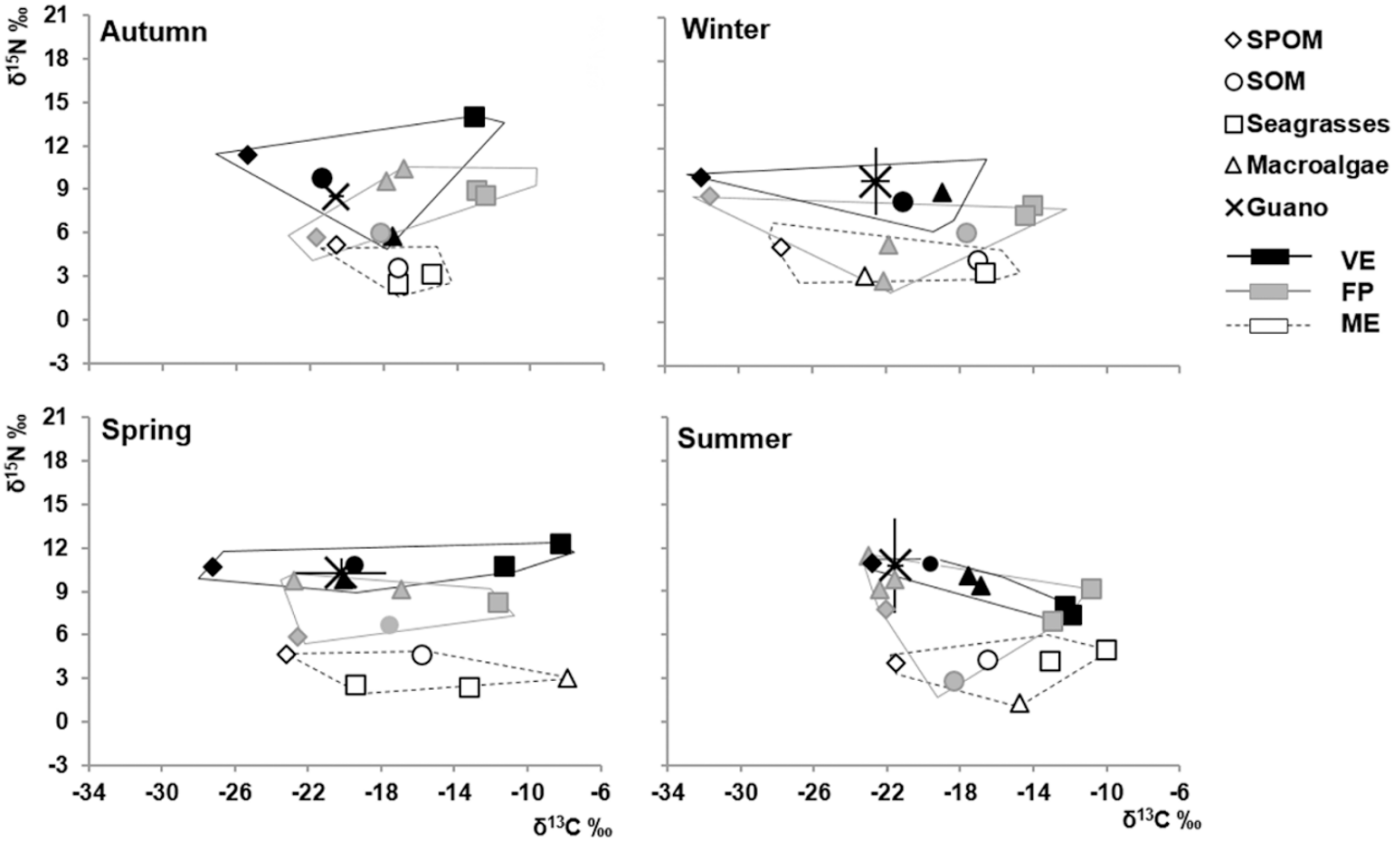
Mean δ^13^C *vs*. δ^15^N (‰) of organic matter sources from the Marinello ponds (VE, FP, ME) across the seasons: suspended particulate organic matter SPOM (diamonds), sedimentary organic matter SOM (circles), seagrasses (squares) and macroalgae (triangles). The isotopic space encompassing the whole variability of organic matter sources in each pond is indicated. The mean isotopic signature (± s.d.) of guano is also superimposed in each graph.

**Fig 3:**
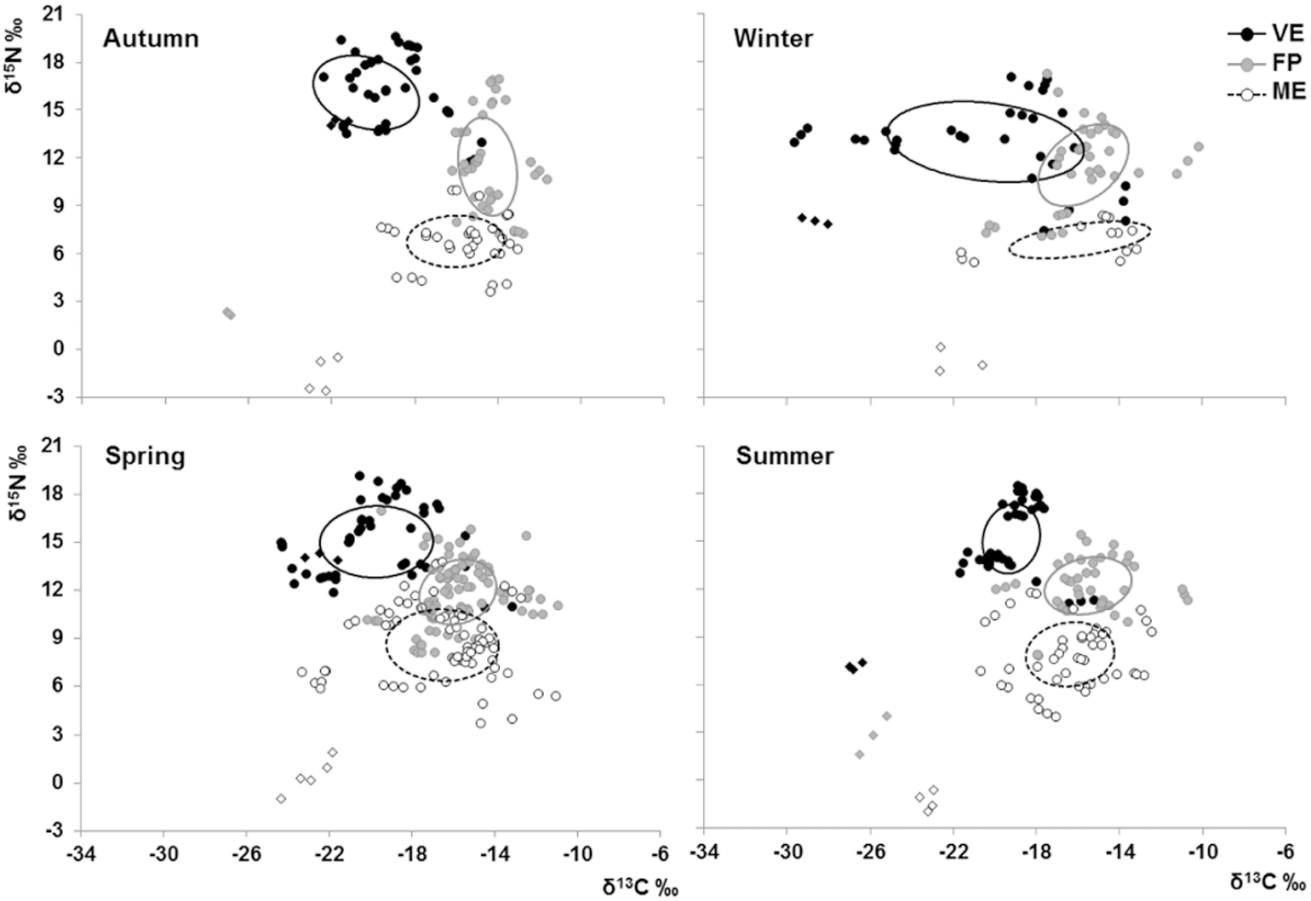
δ^13^C *vs*. δ^15^N (‰) of consumers from the Marinello ponds (VE, FP, ME) across the seasons. Circles enclose the Standard Ellipse Area corrected (SEAc) showing the isotopic niche of consumer communities at each pond and season. Isotopic values of *Loripes lacteus* are shown in the graph (diamonds) but were excluded from the SIBER models, because of the strong ^15^N-depletion.

**Fig 4:**
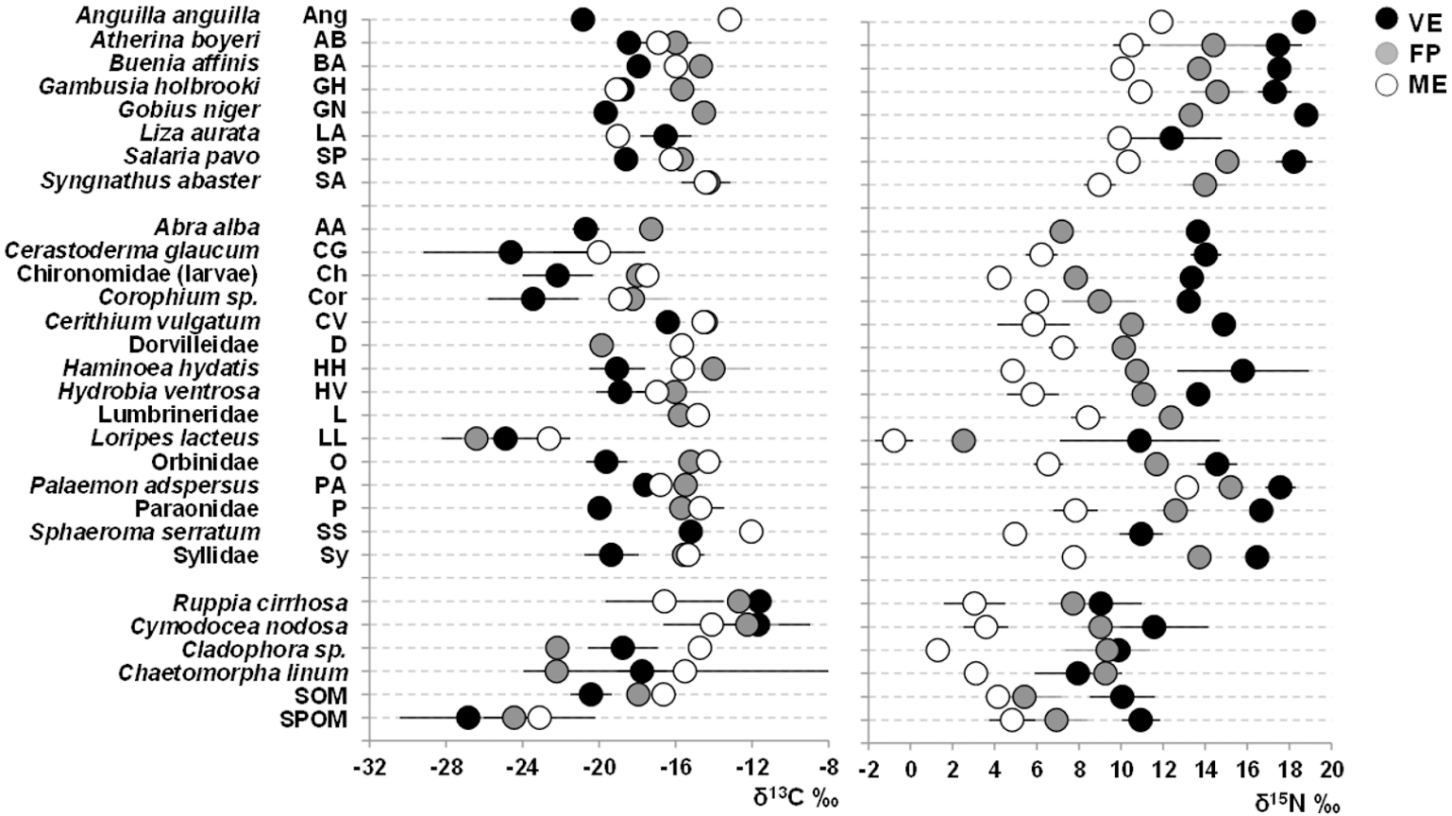
Among-pond variability of δ^13^C and δ^15^N (annual mean ± s.d. ‰) of organic matter sources, invertebrates and fish. Only items common to at least two ponds are shown.

Gull guano isotopic signature varied seasonally, ranging from -23.0 to -18.0‰ and 8.2 to 14.7‰ for δ^13^C and δ^15^N respectively, and was always within the isotopic space encompassing the organic matter sources of VE, the pond receiving the highest amounts of guano (Fig 2). There was no overlap in the isotopic space of organic matter sources between the subsidized- (VE) and non-subsidized pond (ME), while the isotopic space of FP lay in an intermediate position, partially overlapping both VE and ME in all seasons (Fig 2). Likewise, there was a clear variability between ponds as regards consumers: there was only a small overlap of the standard ellipse areas (SEAc), the circle encompassing the isotopic niche of consumers, between VE and FP in winter and between FP and ME in spring (Fig 3). This marked among-pond variability was mainly due to differences in δ^15^N. Organic matter sources and consumers showed a clear ^15^N-enrichment from ME to VE (Fig 4). Mean nitrogen range differences among ponds was fairly high for SPOM and SOM (∼6‰), and even higher for seagrasses and macroalgae (8.0‰ and 8.6‰ for *Cymodocea nodosa* and *Cladophora* sp. respectively), as well as for invertebrates and fish (10.9‰ and 7.9‰ for the snail *Haminoea hydatis* and the blenny *Salaria pavo* respectively). As regards δ^13^C, SOM and SPOM, as well as most consumers (17 out of 21), showed the most depleted values in VE, followed by FP and ME (Fig 4). Mean carbon range differences among ponds were approximately 3.7‰ for both SPOM and SOM. Among invertebrates, they ranged from 2.0 to 5.3‰ (for the gastropod *Cerithium vulgatum* and the polychaete Paraonidae respectively) and, among fish, from 2.5 to 7.7‰ (for *Atherina boyeri* and *Anguilla anguilla*).

Trophic levels estimated in this study did not show remarkable variations among ponds, except for a few species (Table 1). Generally, fish belonged to the third trophic level, with the highest values recorded by *A*. *anguilla*. Among the other fish, *A*. *boyeri*, *Gambusia holbrooki* and *Liza aurata* showed a lower TL in the subsidized pond, VE, than in the others, while *Gobius niger* showed the opposite trend. Overall, invertebrates belonged to the second trophic level, except for the third-trophic-level polychaetes (i.e. Lumbrineridae, Maldanidae, Paraonidae and Syllidae) and the crustacean *Palaemon adspersus*. Differences in TL among ponds were particularly evident for chironomid larvae, *Corophium* sp. and polychaetes Dorvilleidae (Table 1).

**Table 1:** Mean trophic level TL (±s.d.) of invertebrates and fish from the Marinello ponds.

	Ponds		
Invertebrates	VE	FP	ME
*Abra alba*	2.0 (0.1)	0.9	-
*Cerastoderma glaucum*	2.1 (0.3)	-	2.2 (0.5)
*Cerithium vulgatum*	2.3	1.9 (0.2)	2.0 (0.1)
Chironomidae (larvae)	1.9 (0.1)	2.2	1.4
*Corophium* sp	1.9 (0.0)	1.4 (0.5)	1.7
*Dorvilleidae*	-	1.8	2.7 (0.5)
*Gammarus* sp	-	0.9	-
*Haminoea hydatis*	2.7 (0.9)	1.9 (0.1)	1.4
*Holothuria tubulosa*	-	2.2 (0.1)	-
Lumbrineridae	-	2.4 (0.2)	2.9 (0.2)
Maldanidae	-	-	2.9
*Microdeutopus anomalus*	-	0.9	-
*Nassarius reticulatus*	-	2.2 (0.1)	-
Nereididae	-	1.5 (0.0)	-
Orbinidae	2.3 (0.3)	2.2 (0.1)	2.3 (0.2)
*Palaemon adspersus*	3.1 (0.2)	3.1 (0.0)	3.8
Paraonidae	2.9 (0.1)	2.5 (0.3)	2.6 (0.1)
*Phyllodocidae*	-	-	3.1
Sabellidae	-	-	2.6 (0.5)
*Sphaeroma serratum*	1.3 (0.2)	-	1.4
Syllidae	2.8 (0.1)	2.8 (0.0)	2.7 (0.2)
*Venerupis* sp	-	1.6 (0.5)	-
**Fish**			
*Anguilla anguilla*	3.5	-	3.5
*Atherina boyeri*	3.1 (0.4)	3.0 (0.8)	3.4 (0.2)
*Buenia affinis*	3.1	2.8	2.9
*Chelon labrosus*	-	2.6	-
*Gambusia holbrooki*	3.1 (0.1)	3.0 (0.4)	3.3 (0.2)
*Gobius niger*	3.6	2.7	-
*Liza aurata*	1.7 (0.8)	-	2.9
*Salaria pavo*	3.3 (0.2)	3.2	3.0
*Syngnatus abaster*	-	2.9 (0.3)	3.0 (0.3)

Turning to isotopic metrics, in VE, the carbon range of organic matter sources (OM-CR) was wider in spring, while all the other community-wide metrics (C-CR, C-NR, SEAc, CD, MNND, SDNND) showed the highest values in winter and the lowest in summer (Table 2). SDNND had high values also in spring. Unlike VE, the metrics yielded their maximum values overall in winter at FP and in spring at ME. Comparing ponds, annual averaged values of all metrics were higher overall in VE, but similar in FP and ME. C-NR was higher in winter in both FP and VE and in spring in ME, where it markedly dropped in winter due to the paucity of consumers. Food chains were longer in autumn and spring, and shorter in winter and summer in all ponds (Table 2).

**Table 2:** Food web metrics in the Marinello ponds (VE, FP, ME) across the sampling seasons: carbon and nitrogen range of OM sources (OM-CR and OM-NR), carbon and nitrogen range of consumers (C-CR and C-NR), standard ellipse area corrected (SEAc), distance to centroid (CD), mean and standard deviation of the nearest neighbour distance (MNND and SNNDD), food chain length (FCL).

Pond	VE				FP				ME			
Season	Aut	Win	Spr	Sum	Aut	Win	Spr	Sum	Aut	Win	Spr	Sum
**OM-CR**	13.2	14.5	20.7	11.0	11.3	18.0	12.5	12.6	6.1	12.0	16.3	12.1
**OM-NR**	8.9	4.7	3.4	4.5	6.2	6.7	4.5	9.9	3.3	3.8	2.9	5.0
**C-CR**	7.6	16.0	11.2	6.4	4.6	10.2	9.2	9.2	6.5	8.5	12.3	8.3
**C-NR**	7.9	9.7	8.4	7.4	9.7	10.2	8.9	7.6	6.4	2.9	10.1	7.8
**SEAc**	14.7	36.6	19.1	9.5	11.0	16.2	11.6	11.9	9.7	10.5	18.9	13.5
**CD**	2.8	4.7	3.2	2.3	2.9	2.8	2.4	2.4	2.1	2.8	3.0	2.6
**MNND**	0.4	0.8	0.5	0.3	0.4	0.5	0.4	0.4	0.4	0.5	0.4	0.5
**SNNDD**	0.3	0.5	0.5	0.3	0.2	0.3	0.4	0.2	0.2	0.3	0.3	0.3
**FCL**	3.6	2.9	3.6	3.2	3.6	3.2	3.5	3.1	3.6	3.1	3.8	3.4

### Mixing model results

The Bayesian mixing model outcome provided the ranges of possible contributions (95th percentile intervals) of basal organic matter sources to consumers. Among primary consumers, chironomid larvae and C*orophium* sp. exploited mostly SPOM in all seasons in VE, the pond receiving the highest amounts of guano, with a percentage contribution ranging from 15.0 to 62.0% and from 18.0 to 68.0% respectively (S1 Table). In the other ponds, FP and ME, both species showed an overall high consumption of benthic sources, especially SOM and seagrasses, with greater seasonal variability (i.e. C*orophium* sp. in FP).

Although the percentile intervals were wide and often overlapped, indicating a potential use of multiple sources, a greater consumption of SPOM and SOM in *Hydrobia ventrosa* and Orbiniidae polychaetes in VE was observed in summer and autumn, their contribution ranging from 10.0 to 56.0% (SPOM) and from 0.0 to 52.0% (SOM). In contrast, in the other ponds, *H*. *ventrosa* showed a mixed use of all sources in all seasons, except for the summer peak of seagrass percentage contribution (18.0–72.0% and 13.0–77.0% in FP and ME respectively), while Orbiniidae greatly exploited seagrasses in all seasons in FP (22.0–86.9%) and only in autumn in ME (16.0–90.0%).

The role of basal sources for secondary consumers mirrored the pond- and season-specific patterns highlighted in primary consumers. In detail, in the subsidized pond, the pathway leading to Paraonidae polychaetes was based on SPOM and SOM in all seasons (12.0–57.0% and 0.0–57.0% respectively), while all basal sources contributed to the pathway leading to the shrimp *Palaemon adspersus* and both fish (*Atherina boyeri* and *Salaria pavo*), particularly in spring and summer. Secondary consumers relied mainly on seagrasses in FP and on both SOM and seagrasses in ME.

**Fig 5:**
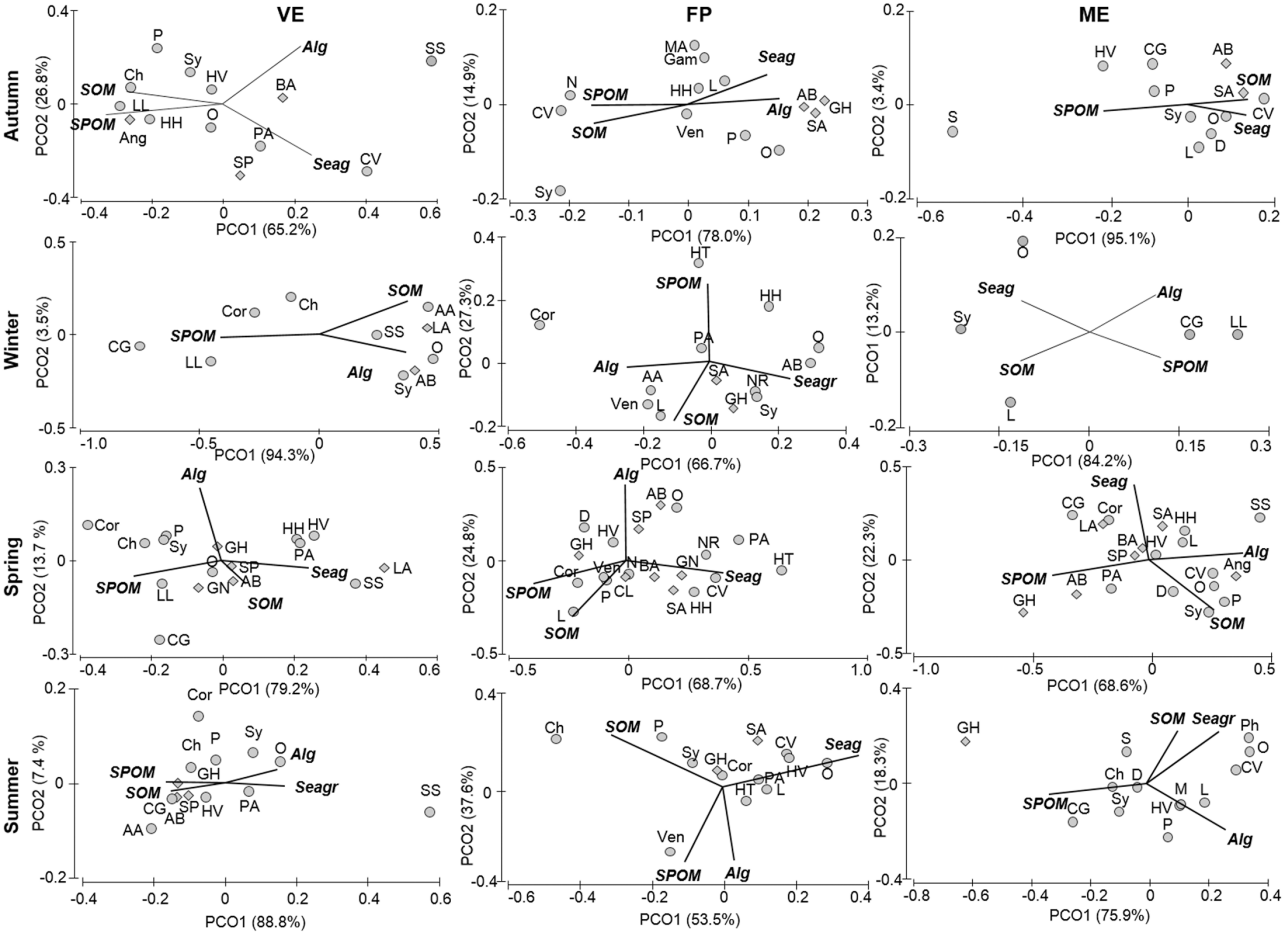
Principal coordinate analysis (PCO) representing the contribution of organic matter sources to the consumers’ diet in the Marinello ponds (VE, FP, ME, in columns) across the sampling seasons (in rows). Minimum, maximum (expressed as 95th percentiles) and mean contribution, estimated using SIAR, were used as variables. The vectors corresponding to the mean contribution of OM sources were superimposed on each graph: sedimentary organic matter (SOM), suspended particulate organic matter (SPOM), macroalgae (Alg), seagrasses (Seag). Acronyms of consumers are the same as in Fig 4. Invertebrates are represented with circles, fish with diamonds.

These pathways were confirmed at a multivariate level when assessing the role of organic matter sources by principal coordinate analysis (PCO) on the Bayesian mixing model outcome. Despite the overall high variability in the relative contributions of the four organic matter sources (SPOM, SOM, macroalgae and seagrasses) within food webs, PCO was as an effective method for representing graphically the prevailing organic matter pathways characterising the food web, the consumers being distributed in the ordination based on the contribution of the basal sources. Axis 1 always explained a substantial percentage of the total variation, from 53.8% to 95.1%, while the variability explained by axis 2 was in the range 3.4–37.6% (Fig 5). In VE, the subsidized pond, most species were close to the vectors of SPOM and SOM, especially in summer and autumn, suggesting that the biotic community relies largely on these basal sources. The importance of the pathway based on SPOM decreased in spring and above all in winter, as most consumer positions shifted towards other sources (SOM and macroalgae in winter and SOM and seagrasses in spring). In FP, the pond adjacent to VE, benthic sources, especially seagrasses, were dominant at the base of the food web throughout the year. Similarly, seagrasses, as well as SOM, were the most important basal sources in the furthest pond, ME. In spring only, in both FP and ME, the greater spatial dispersion of consumers in the ordination indicated a trophic reliance on all sources.

## Discussion

### Seabird-induced isotopic shift in basal organic matter sources and consumers

δ^15^N of organic matter sources and consumers increased markedly along the gradient of avian input, with the highest values in VE, the subsidized pond. δ^15^N has been used as a proxy for ornithogenic origin of nutrients in various aquatic systems [7,19], including Marinello ponds [9], because the typical ^15^N-enriched signature of excreted guano [18]. Due to the resident habit of the Marinello gull colony, VE pond is subject to constant guano input, both directly by flying gulls and indirectly by runoff from the cliff above. Ornithogenic nitrogen is consequently an important part of the nitrogen pool in VE, triggering great isotopic enrichment of all basal sources. Consumers were also distinctly ^15^N-enriched, providing evidence that ornithogenic nitrogen flowed throughout the food web. Despite the low density and nutrient load of the Marinello gull colony [9], the isotopic enrichment in organisms was even more pronounced than in biota living close to high density colonies [21]. This may be due to the particular hydrological features of this semi-enclosed ecosystem (e.g. scarce water exchange, high bentho-pelagic coupling) exacerbating the guano–derived effects.

Differently, δ^13^C is not usually considered an indicator of avian influence [8]. Nevertheless, the good match between δ^13^C of guano and SOM in VE suggests accumulation of seabird-derived organic matter, as only a small fractionation of avian-derived carbon after guano deposition was reported in the literature [10,19,33]. Gull guano affects water column trophic status and primary production in VE [9], However, SPOM δ^13^C was lower than that of guano and showed strong seasonal variability. Terrestrial organic matter is typically ^13^C-depleted compared to marine organic matter [34]. Thus, runoff of terrigenous material from the cliff into the subsidized pond, which is especially abundant during winter rainfall when guano input is at its minimum, is likely responsible for the spatial and seasonal variability of the SPOM isotopic signature.

### Food web structure in the subsidized pond

Different guano-derived nutrient subsidies influenced the trophic structure of the Marinello ponds. Overall in VE, the pond subject to the highest guano input, isotopic metrics proxies for total and average trophic diversity (SEAc and CD) [27,28] and for trophic redundancy (MNND and SDNND) [27] showed a distinct seasonal pattern opposite to that of guano, peaking in winter when guano was at its minimum and vice versa in summer. Indeed, trophic status and phytoplanktonic productivity are higher in the seasons characterised by highest guanotrophication (late summer and autumn), than in winter, when the lowest guanotrophication occurs, and in spring, when productivity starts to increase [9]. Consistent with these findings, current results highlighted that in summer, the food web relies chiefly on the planktonic pathway, and consequently the isotopic niche reduces and trophic redundancy increases. In contrast, when productivity is lower, primary consumers tend to switch to other, more available, sources, with a consequent enlargement of the isotopic niche and decrease in species packing. Therefore, consistently with the literature [4,5], when guano input was low to moderate, consumers exhibited a higher trophic diversity and complexity, benefiting from the co-existence of parallel pathways based on subsidized planktonic and benthic basal sources. On the other hand, in summer, when guano input increased maximising its trophic effects, lower trophic diversity, higher redundancy and simplification of food webs was evident.

Food chain length (FCL) was fairly similar among ponds, with the lowest values in winter and the highest in spring, similarly to other Mediterranean lagoons under varying degrees of eutrophication [17]. FCL is an important ecosystem attribute that integrates the assimilation of energy through all the trophic pathways leading to top predators and plays a central role in determining ecosystem functioning. FCL is influenced by the combined effect of several processes: it is constrained by ecosystem size and frequent and intense disturbance, but favoured by high resource availability and productivity [35,36]. In this area, while enhancing primary productivity, guano input causes harsh environmental conditions in VE and acts as a disturbance driver [13]. Therefore, in VE, the potentially positive effect of increased primary productivity on FCL seems to be hampered by disturbance, resulting in the overall uniformity of FCL in the similar-sized Marinello ponds.

### Organic matter pathways and trophic switch of consumers

Bayesian mixing models revealed that organic matter sources played a different role in the food webs of the Marinello ponds. SPOM and SOM, basically composed of living and freshly deposited phytoplankton [9], overall fuelled the biotic communities in VE, the pond subject to high nutrient subsidy. Seagrasses and SOM were the most important organic matter sources in the other two ponds, FP and ME. As regards seasonal variability of trophic pathways, with the exception of FP, where the benthic pathway was dominant throughout the seasons, the planktonic pathway was less important in VE mainly in winter, and the benthic pathway was less important in ME only in spring. Phytoplankton blooms in lagoons are typically long-lasting, from spring to summer [37], and supply high quality organic matter to the seabed, which fuels benthic communities, favouring bentho-pelagic coupling. Accordingly, the avian-induced excessive phytoplankton production in VE and the consequent sinking of large amounts of phytodetritus [9] prolonged the planktonic pathway until autumn, while it was restricted to only spring in ME where guano input does not occur. On the other hand, when phytoplanktonic production rates naturally decrease as an effect of reduced temperature (i.e. winter), planktonic sources became less available to primary consumers, which consequently shifted towards benthic food resources in VE. Indeed, planktonic and benthic pathways were recently considered alternative stable states between which coastal systems may shift depending upon external conditions, chiefly temperature and nutrient availability [38]. Thus, it can be inferred that the ornithogenic nutrient subsidy in VE had a marked impact on organic matter pathways and food webs, by inducing the coexistence of parallel pathways at moderate input, and a marked erosion of the benthic pathway in favour of the planktonic at higher input.

Looking at primary consumers, deposit feeders are the most abundant trophic group and are dominated by chironomid larvae, *Corophium* sp., *Hydrobia ventrosa* and the polychaete Orbiniidae [13]. Chironomid larvae and the amphipod *Corophium* sp. consumed mainly SPOM in the guanotrophic pond throughout the year, while they generally switched to benthic sources, mainly SOM and seagrasses, in the other ponds. Chironomid larvae have been shown to selectively assimilate phytoplankton as fresh deposits from bulk sediments and shift to SOM and benthic diatoms as benthic production increases [39], while *Corophium* spp. have generally been described as burrowing deposit feeders that select diatoms and bacteria from sediment [40]. The continuous exploitation of SPOM in VE is evidently favoured by guanotrophication, which implies a high and long-lasting production of high quality organic matter (phytoplankton) [3] and its sinking onto the sediment. In contrast, the consumption of benthic sources observed in the other ponds may reasonably be linked to their greater availability.

*Hydrobia ventrosa* and the polychaete Orbiniidae generally showed a mixed use of basal sources in all ponds. However, SPOM and SOM consumption were still important in VE, especially in summer and autumn, while in the other two ponds peaks of seagrass consumption were also evident. These two species are considered both opportunistic and unselective deposit feeders [40,41], and their diet reflected the organic matter source availability in the ponds.

Turning to secondary consumers, the polychaete Paraonidae varied its trophic level from 2.5 to 2.9, consistent with an unselective omnivorous diet in all ponds. The lower TL of *Palaemon adspersus* and *Atherina boyeri* in VE than in ME, also observed in the other fish *Gambusia holbrooki* and *Liza aurata*, may reveal trophic plasticity with a more omnivorous regime in VE, as an adaptive response to guanotrophication, consistent with both theoretical [42] and empirical studies [17]. Furthermore, the mixed consumption of benthic and pelagic basal sources, highlighted in predators from VE, suggests they couple the coexisting parallel planktonic and benthic pathways. This is a common feature in mobile predators in aquatic systems [43]; lagoon species in particular, which are adapted to variable conditions, exhibit flexible and opportunistic feeding habits and often undergo diet shifts, mirroring local prey availability and diversity [44].

## Conclusions

In focusing on the ecological response of coastal ponds to guano input, this study has pinpointed a marked ^15^N enrichment of the subsidized biota, confirming that seabird-derived nutrients were recycled in the planktonic and benthic compartments across trophic levels. The overall food web response to guanotrophication was a long-lasting erosion of the benthic pathway in favour of the planktonic one, especially when guano input was higher. Primary consumers, mostly generalist deposit feeders, showed consistent trophic switches as an adaptation to the differing availability, and presumably nutritional quality, of organic matter sources. Secondary consumers had a more omnivorous feeding regime and triggered the coupling of planktonic and benthic pathways in the subsidized pond. Community-trophic niche width varied according to guanotrophication seasonality: it narrowed and widened when guanotrophication increased and decreased respectively. In contrast, the similarity of food chain length among all ponds may be due to the opposite effect of disturbance due to intense guanotrophication and increased primary productivity, rather than to homogeneity in the ecosystem processes among ponds.

To conclude, assessment of food web structure and metrics revealed different ecosystem processes and dynamics between ponds, as an effect of the differing ornithogenic subsidies. In particular, seasonal variability in the isotopic metrics was associated with changes in guanotrophication, indicating a detrimental condition when the trophic effects of guano input intensify.

Lastly, the study of a food web influenced by continuous seabird nutrient subsidies contributes to a better understanding of the potential role of seabirds in aquatic ecosystems, an issue which has until now been overlooked.

## Supporting Information

S1 Table Bayesian mixing model output indicating the percentage contribution of organic matter sources to the diet of dominant primary and secondary consumers in the Marinello ponds (VE, FP, ME) across the sampling seasons. Low: lower 95th percentile proportion; High: higher 95th percentile proportion.
(DOCX)
