# Supplementary material for: Guano-Derived Nutrient Subsidies Drive Food Web Structure in Coastal Ponds
Source: PLoS One. 2016 Mar 8;11(3):e0151018. doi: 10.1371/journal.pone.0151018 (PMC4783068; doi:10.1371/journal.pone.0151018)
Supplement: S1 Table — Low: lower 95th percentile proportion; High: higher 95th percentile proportion. (DOCX) [file pone.0151018.s001.docx]

Supplemental Information

Appendix 1: Bayesian mixing model output indicating the percentage contribution of organic matter sources to the diet of dominant primary and secondary consumers in the Marinello ponds (VE, FP, ME) across the sampling seasons. Low: lower 95th percentile proportion; High: higher 95th percentile proportion.
